# Accuracy of automated intracerebral hemorrhage volume measurement on non-contrast computed tomography: a Swedish Stroke Register cohort study

**DOI:** 10.1007/s00234-022-03075-9

**Published:** 2022-11-03

**Authors:** Amir Hillal, Gabriella Sultani, Birgitta Ramgren, Bo Norrving, Johan Wassélius, Teresa Ullberg

**Affiliations:** 1 Medical Imaging and Physiology, Skåne University Hospital, 221 85 Lund, Sweden; 2 Department of Clinical Sciences Lund, Lund University, 221 85 Lund, Sweden; 3 Department of Neurology, Skåne University Hospital, 205 02 Malmö, Sweden

**Keywords:** Intracerebral hemorrhage, Non-contrast computed tomography, Brain, Automated image analysis software

## Abstract

**Purpose:**

Hematoma volume is the strongest predictor of patient outcome after intracerebral hemorrhage (ICH). The aim of this study was to validate novel fully automated software for quantification of ICH volume on non-contrast computed tomography (CT).

**Methods:**

The population was defined from the Swedish Stroke Register (RS) and included all patients with an ICH diagnosis during 2016–2019 in Region Skåne. Hemorrhage volume on their initial head CT was measured using ABC/2 and manual segmentation (Sectra IDS7 volume measurement tool) and the automated volume quantification tool (qER–NCCT) by Qure.ai. The first 500 were examined by two independent readers.

**Results:**

A total of 1649 ICH patients were included. The qER–NCCT had 97% sensitivity in identifying ICH. In total, there was excellent agreement between volumetric measurements of ICH volumes by qER–NCCT and manual segmentation by interclass correlation (*ICC* = 0.96), and good agreement (*ICC* = 0.86) between qER–NCCT and ABC/2 method. The qER–NCCT showed volume underestimation, mainly in large (> 30 ml) heterogenous hemorrhages. Interrater agreement by (*ICC*) was 0.996 (95% *CI*: 0.99–1.00) for manual segmentation.

**Conclusion:**

Our study showed excellent agreement in volume quantification between the fully automated software qER–NCCT and manual segmentation of ICH on NCCT. The qER–NCCT would be an important additive tool by aiding in early diagnostics and prognostication for patients with ICH and in provide volumetry on a population-wide level. Further refinement of the software should address the underestimation of ICH volume seen in a portion of large, heterogenous, irregularly shaped ICHs.

**Supplementary Information:**

The online version contains supplementary material available at 10.1007/s00234-022-03075-9.

## Introduction

Spontaneous intracerebral hemorrhage (ICH) accounts for up to 30% of all acute strokes worldwide with a 30-day mortality rate up to 40% [1]. Non-contrast computed tomography (NCCT) is the most widely used neuroimaging modality for rapid and accurate diagnosis of ICH and assessment of the hematoma volume which is considered the single strongest predictor of outcome for ICH patients [2]. Studies have shown that a baseline hematoma volume of 30 ml or more is associated with hematoma expansion and poor outcome, whereas a hematoma volume of 10 ml or less is associated with a lower probability of hematoma expansion and predict a favorable functional outcome [3–5].

Several methods can be used to measure ICH volume on NCCT. One of the most common methods is the ABC/2 method which assumes an ellipsoid hematoma shape and therefore may over- or under-estimate the volume of irregularly shaped hematomas [6–9]. The ABC/2 method is therefore best used to measure regular-shaped parenchymal ICH that does not include an intraventricular hemorrhage (IVH) component. Since ICH with IVH is in itself a negative outcome predictor [3, 4] due to its complex pathophysiology including a more extensive blood distribution within the brain and more complex clearing mechanisms [10]. Later methods include computer-assisted manual segmentation and volume measurement that are more accurate also for irregularly shaped hematomas, but labor intensive and therefore not routinely used in clinical care [11–13].

Recently, automated image analysis software based on deep learning algorithms have been developed for detection and volume quantification of ICH [14–18]. Implementing such automated imaging analysis tools in routine healthcare may improve early detection by prioritizing among radiological exams and reduce missed ICH diagnoses. Routine use of automated volume segmentation tools may also aid in the early prognostication. However, the benefit of automated volumetric segmentation tools hinges on its accuracy.

The aim of this study was to validate recently developed automated image analysis software (qER–NCCT) for volumetric measurement of ICH volume on NCCT in a large regional population of patients with ICH identified in the Swedish Stroke Register (Riksstroke).

## Methods

### Study design

We performed a retrospective observational study based on the Swedish quality register for stroke care, Riksstroke. Patients were included for analysis if they had NCCT images from the date of stroke onset available in the regional picture archiving and communication system (PACS).

The Swedish Ethical Review Authority approved the study (#2020–06,800) and waived informed consent. Furthermore, all patients registered in Riksstroke are informed of registration and handling of patient data for research purposes.

### Participants

#### Inclusion criteria

All patients > 18 years within the Skåne region (1.5 million inhabitants out of the 10 million inhabitants in Sweden), registered in Riksstroke during 2016–2019 with spontaneous non-traumatic intracerebral hemorrhage (ICD-10 I.61). Spontaneous ICH caused by presumed deep perforator vasculopathy or cerebral amyloid angiopathy (CAA), vascular malformation, dural sinus thrombosis, complications to reperfusion treatments or brain surgery or other rare conditions were included.

#### Exclusion criteria

Patients with ICH caused by trauma or underlying brain tumor/metastasis were excluded. Patients lacking CT images from the date of presentation or lacking pre-operative images were excluded, as were patients whose CT-images had severe technical artifacts due to for example motion artefacts.

### Data sources

#### The Swedish Stroke Register — Riksstroke

Riksstroke is the Swedish quality register for stroke care covering > 90% of Swedish hospitalized stroke patients. Data are entered in Riksstroke by dedicated personnel.

##### Image data

Patients were included in the final analysis if they had NCCT images from the date of presentation at the hospital. The Skåne region has a common PACS system where imaging data from all hospitals (*n* = 13) in the region are collected, thus ensuring access to all available imaging data from all hospitals providing stroke care within the region. Neurosurgery patients, however, may be transferred from outside the region, thereby lacking pre-operative image data which is only stored in the regional PACS for 90 days following image transfer. In total, images were lacking in 9 patients and affected by severe artifacts in 1 patient.

The included head CT scans were performed on scanners from all major manufacturers, and thin axial reconstructions used for the analyses had a slice thickness of 0.5–1 mm.

##### Image evaluation

NCCT scans for all included (1649) patients were examined and evaluated by a radiology resident with 1 year of neuroradiology experience. The initial 500 scans were also independently evaluated by a senior neuroradiologist with more than 20 years’ experience for interrater agreement assessment. Prior to image evaluation the readers made consensus reading with the senior members of the scientific team of > 50 other ICH cases to ensure an even standard of assessment. The readers did not have access to the automated image evaluation by qER–NCCT or to the evaluation results by the other reader at the time of their assessments. The following imaging findings were noted: presence of ICH (yes/no), location (lobar and/or deep ICH or only IVH), side (right, left), single or multifocal (defined as multiple ICHs without any connection), presence of subarachnoid component (yes/no), presence of fingerlike projections (yes/no), presence of subdural component (yes/no), presence of intraventricular extension (yes/no), presence of midline shift (yes/no, if yes: midline shift measured in mm) or hydrocephalus ( yes/no) [19], presence of known or newly diagnosed vascular malformation at the time of stroke onset (yes/no).

##### Volume quantification

Hematoma volumes were measured using three different volumetric methods:

(i) Manual segmentation of all ICH;
(ii) Fully automated segmentation of all ICH; and
(iii) ABC/2 method for parenchymal ICH without IVH

Manual segmentation was done using the Sectra Volume Measurement tool (Sectra IDS7, Sectra, Linköping, Sweden). The fully automated volume measurement was done using the qER–NCCT volume quantification tool (Qure.ai, Mumbai, India). In isolated parenchymal ICH the ABC/2 method was considered ground truth in comparison with qER–NCCT, whereas in the comparison between manual segmentation and qER–NCCT, which was done for all bleeds, manual segmentation was considered ground truth.

#### ABC/2 method

In the ABC/2 method (also known as the TADA formula), A is the largest diameter of the hematoma on axial images; B is the largest diameter perpendicular to A on the same image slice, and C is the number of slices in which the hematoma is seen, multiplied by the slice thickness [20]. All lengths were registered in millimeters (mm) and volumes in milliliters (ml).

For intracerebral hemorrhages with an intraventricular extension, only the parenchymal component was measured by the ABC/2, since this was the original indication for the measurement [6–9].

#### Manual segmentation

Sectra Volume Measurement tool (Sectra IDS7, Sectra, Linköping, Sweden) is a manual segmentation tool where an initial line is manually drawn between two margins of the hematoma, and the software identifies the margins of the entire hematoma and calculates the volume. For additional hematoma components, additional lines can be added to calculate the total volume.

#### qER–NCCT

The automatic segmentation tool (qER–NCCT) is developed using deep learning methods trained primarily to identify hyperdense (acute) intracranial hemorrhages on thin slice axial plan DICOM images. The device is intended to assist trained medical specialists by indicating the presence of the following findings on NCCT head CT scan images: intracranial hemorrhage, mass effect, midline shift, cranial fracture, infarcts, and cerebral atrophy. The device can also quantify and outline the abovementioned pathologies. For this study, we turned off all features except hyperdense (acute) intracranial bleeds. The qER–NCCT tool was used according to the instructions for use, and all images were processed by the software without any pre-processing of the images.

Thin slice axial plane DICOM images for all included patients were sent from PACS to the qER–NCCT software via a teleradiological transfer function within the IDS7 application. The qER–NCCT program was installed separate from the IDS7 PACS on a local server within the hospital firewall. The software automatically returned DICOM images to PACS including a copy of the thin sections with the margins of the identified acute ICH volume outlined and a standardized report including identification of ICH (yes/no) and the total volume of the hematoma(s) measured in ml.

### Statistical analysis

IBM SPSS statistics version 25 was used for all statistical analyses. Demographic data and ICH measurements were analyzed using standard descriptive statistics. One-sample *t*-tests were done to obtain the mean, standard deviation and mean difference between the volume segmentation methods and between the two raters as well.

Bland–Altman plots were drawn to assess the agreement between the different volume segmentation methods and the interrater agreement, and the limits of agreement for the volumetric measurements were calculated. Intraclass correlation coefficient (*ICC*) was also calculated to determine the agreement between the volumetric measurement methods as well as between both raters. Interpretation of *ICC* was done according to Koo and Li (< 0.5 — poor; 0.5–0.75 — moderate; 0.75–0.9 — good; and > 0.9 — excellent agreement) [22].

## Results

**Fig. 1:**
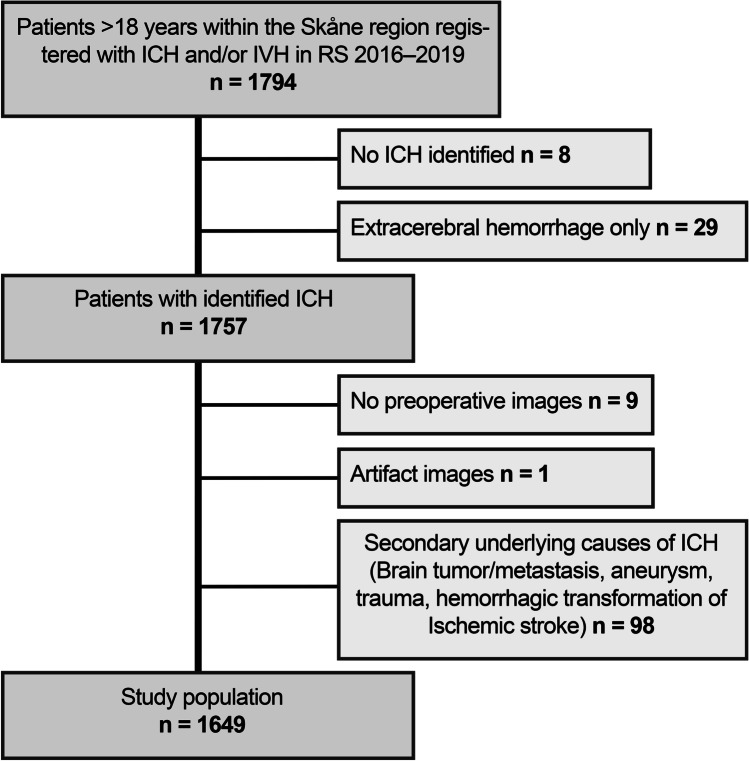
A consort diagram showing the different exclusion criteria in establishing the final study population of 1649 patients

The population obtained from Riksstroke and the number of patients excluded for each exclusion criteria, resulting in a final study population of 1649 patients, is shown in Fig. 1. The median age was 76 years (range 18–102 years) and 46% were women. Detailed ICH characteristics are shown in Table 1.

**Table 1:** ICH characteristics and for the study population.

	Number of patients(*n*** = **1649)	Percentage
ICH characteristics		
Multifocal	107	6.5%
Finger-like projections	198	12%
Subarachnoid component	299	18.1%
Intraventricular extension	719	43.6%
ICH location		
Supratentorial	1399	84.8%
Lobar	662	40.1%
Deep	734	44.5%
Both lobar and deep	4	0.2%
Infratentorial	202	12.3%
Brainstem	72	4.4%
Cerebellum	129	7.8%
Both brainstem and cerebellar	1	0.1%
Intraventricular hemorrhage without parenchymal hemorrhage	48	2.9%

*ICH* Intracerebral hemorrhage

**Fig. 2:**
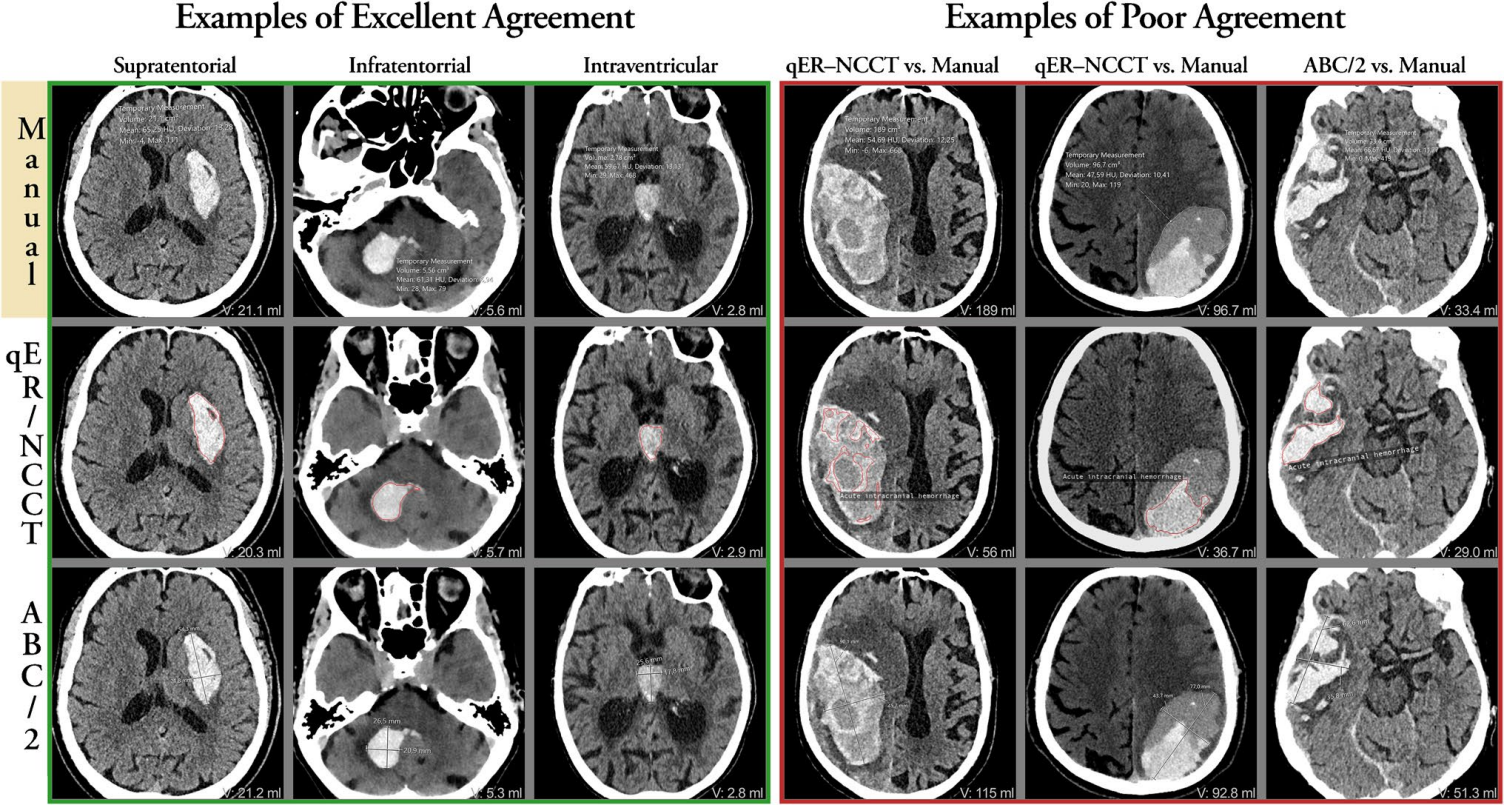
The top row shows the outline of the manual segmentation (gold standard); the middle row shows the outline of the qER–NCCT software; and the bottom row shows the A and B diameters of the ABC/2 measurement method. The volume of each measurement is shown in the bottom right corner of each image. The left 3 columns show typical cases with excellent agreement with supra-, infratentorial, and intraventricular locations, respectively. The fourth and fifth columns illustrate two typical cases of large heterogenous lobar ICH where the qER–NCCT software has delineated only the most hyperintense portions of the hematomas, thereby underestimating the total ICH volume. The sixth column illustrates a heterogenously shaped ICH where the ABC/2-method overestimates the hematoma volume, whereas the agreement between the qER–NCCT software and manual segmentation is excellent

**Fig. 3:**
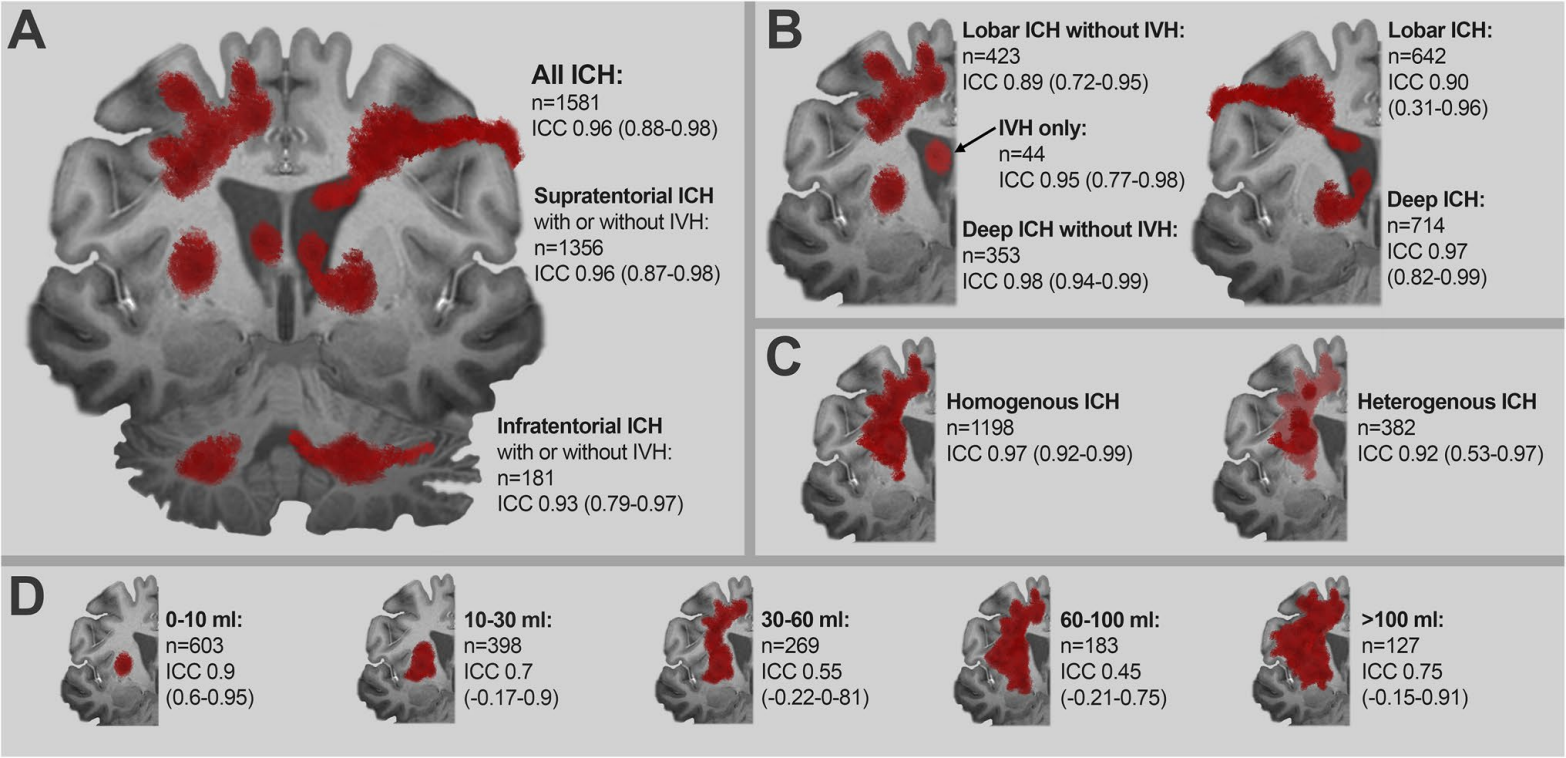
Illustration of the agreement between manual segmentation (gold standard) and the qER–NCCT software as measured by ICC for the various ICH subgroups. Panel **A** shows the agreement for all ICH and for supra- and infratentorial ICH separately. Panel **B** shows the agreement for lobar and deep supratentorial ICH. The left part shows the agreement for lobar and deep ICH without IVH and for IVH only. The right part shows the agreement for all lobar and deep ICH, regardless of IVH or not. Panel **C** shows the agreement for homogenous ICH and heterogenous ICH separately. Panel **D** shows the agreement grouped for different ICH volumes

The automated qER–NCCT/Quant software was technically successful in returning a result to the PACS in 1638 of 1649 cases (99.3%). The software had 97% sensitivity for identifying ICH on NCCT. There were a total of 57 false-negative ICHs, of which 54 were ≤ 1 mm in diameter. Image examples are shown in Fig. 2. The agreement between manual segmentation and qER–NCCT volume measurement, according to ICC for all ICH and separated in subgroups, is shown in Fig. 3.

### Interrater agreement

The mean of volumes obtained by the two independent raters for manual segmentation were 34.0 and 34.3 ml, respectively, and the difference was not statistically significant (*MD* = 0.3, *t* = -1.3, *p* = 0.196). The *ICCs* of manual segmentation between the two raters were 0.996 (95% *CI*: 0.99–1.00).

The mean of volumes obtained by the two independent raters using the ABC/2 method were 16.6 and 16.3 ml, respectively, and the difference was not statistically significant (*MD* = 0.3, *t* = 0.84, *p* = 0.4). The *ICCs* of ABC/2 between the two raters were 0.98 (95% *CI*: 0.96–0.99).

The interrater agreement (Supplementary Table 1) by ABC/2 and manual segmentation are shown in Bland–Altman plots (Supplementary Fig. 1).

### Agreement between manual segmentation and the qER–NCCT volume for all patients

**Fig. 4:**
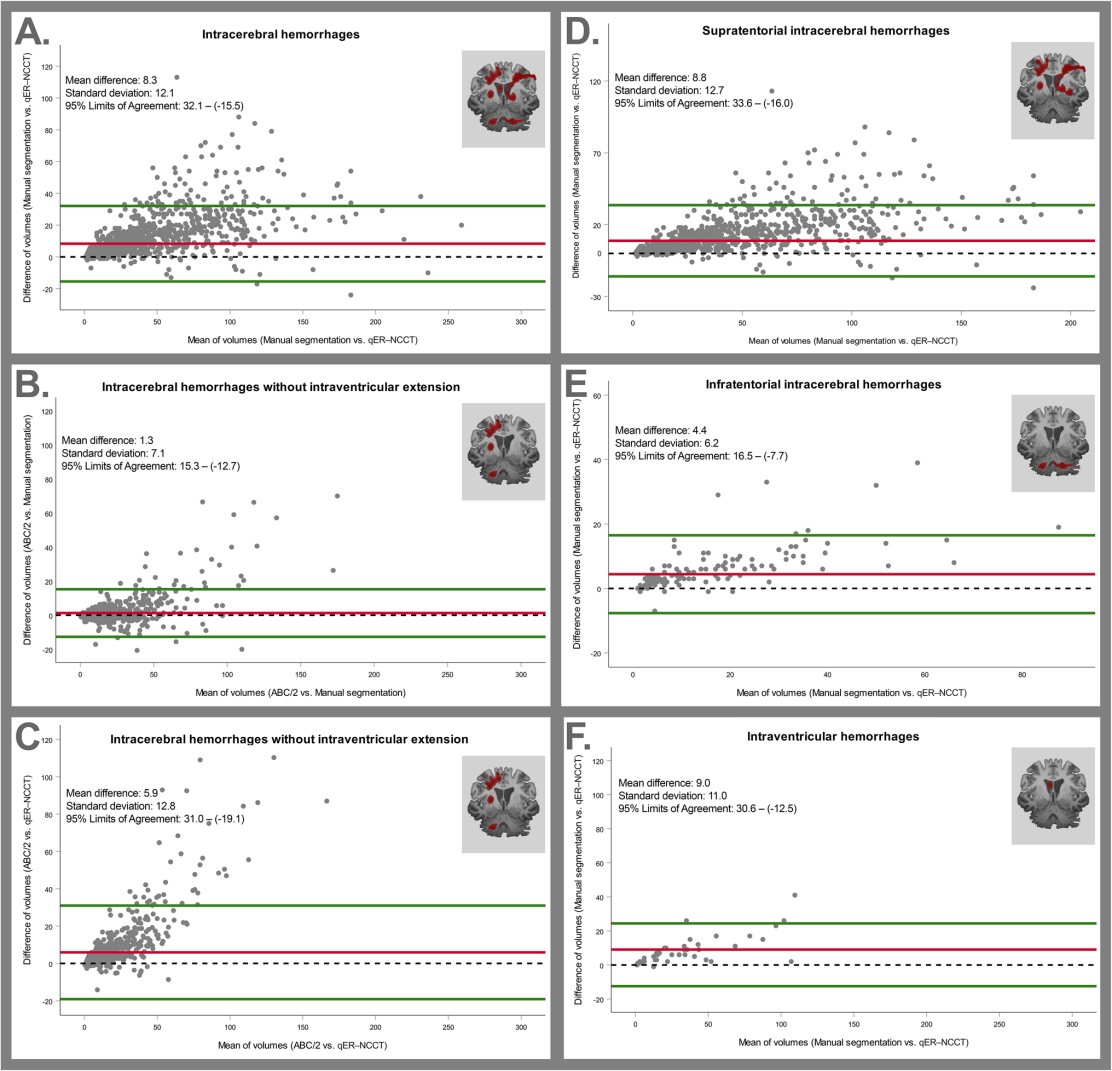
Top row shows Bland–Altman plots comparing the different volumetric measurement methods (manual segmentation, the qER–NCCT software and the ABC/2 method). Panel **A** shows the agreement between manual segmentation (gold standard) and the qER–NCCT software for all ICH. Panel **B** shows the agreement between manual segmentation (gold standard) and the ABC/2 method for ICH without IVH. Panel **C** shows the agreement between the ABC/2 method (gold standard) and the qER–NCCT software for ICH without IVH. Bottom row shows Bland–Altman plots for the agreement between manual segmentation (gold standard) and the qER–NCCT software for all supratentorial ICH (panel **D**), all infratentorial ICH (panel **E**) and for IVH only (panel **F**), respectively. The mean differences, the standard deviation, and 95% limits of agreement are shown in each plot

Of the 1649 patients, qER–NCCT completed a volumetric measurement in 1581 cases that were included in this analysis. There was excellent agreement between the two methods with an *ICC* of 0.96 (95% *CI*: 0.88–0.98) (Table 2 and Fig. 3). The Bland–Altman plot (Table 2 and Fig. 4) showed a statistically significant (*p* < 0.01) difference of volumes between the two methods comprising increased difference with increasing ICH volume. The mean difference (MD) of qER–NCCT compared to manual segmentation was 8.3 ml and the 95% limits of agreement were (− 15–32), as shown in Table 2. The differences were mainly explained by volume underestimation of the qER–NCCT tool which was mostly seen in large ICHs (> 30 ml) with heterogenous attenuation (see examples in Fig. 2). Overestimation by the qER–NCCT tool was rare, seen only in 37 (2.3%) cases. The difference in volume was 2 ml or less in 41% of all cases, of which 93% had homogenous attenuation; 83% were small ICH (≤ 10 ml), and 80% had no IVH extension.

**Table 2:** Panel A: agreement between manual segmentation (considered gold standard) and volume measurement by qER–NCCT for all ICH (left column) and for IVH only (right column). Panel B: agreement between manual segmentation (considered gold standard) and volume measurement by qER–NCCT (left column), the ABC/2 method and qER–NCCT (middle column), and manual segmentation, and the ABC/2 method (right column) for ICH without IVH extension. Panel C: agreement between manual segmentation (considered gold standard) and volume measurement by qER–NCCT, depending on ICH size and attenuation.

Panel A: agreement for all ICH and IVH only				
Manual segmentation vs. qER–NCCT				
Agreement statistics	Manual vs. qER–NCCT		Manual vs. qER–NCCT	
	*n* = 1581 (all ICH)		*n* = 44 (IVH only)	
Mean difference (ml)	8.3		9.0	
Standard deviation (ml)	12.1		11.0	
Median difference (ml)	4.0		6.0	
*IQR* (ml)	10.0		9.0	
95% *LoA* (ml, low–high)	− 15.0^__^32.0		− 12.0^__^30.6	
*ICC*	0.96		0.95	
95% *CI*	(0.88–0.98)		(0.77–0.98)	
Panel B: agreement for ICH without IVH extension				
Manual segmentation vs. qER–NCCT, ABC/2 vs qER–NCCT and ABC/2 vs. Manual segmentation				
Agreement statistics	Manual vs. qER–NCCT	ABC/2 vs. qER–NCCT		ABC/2 vs. Manual
	*n* = 836	*n* = 836		*n* = 891
Mean difference (ml)	4.5	5.9		1.3
Standard deviation (ml)	8.5	12.8		7.1
Median difference (ml)	2.0	1.5		-0.1
*IQR* (ml)	5.0	5.9		1.8
95% *LoA* (ml, low–high)	− 15.0^__^32.0	− 12.0^__^30.6		− 12.7^__^15.3
*ICC*	0.92	0.86		0.97
95% *CI*	(0.84–0.95)	(0.78–0.90)		(0.97–0.98)
Panel C: agreement depending on size and heterogenicity for all ICH				
Manual segmentation vs. qER–NCCT				
Agreement statistics	Number of patients	Mean difference		*ICC* (95% *CI*: upper, lower)
ICH volume < 10 ml	603	1.0		0.90 (0.60–0.95)
ICH volume (10–30 ml)	398	4.9		0.70 (− 0.17–0.9)
ICH volume (30–60 ml)	269	11.0		0.55 (− 0.22–0–81)
ICH volume (60–100 ml)	183	20.0		0.45 (− 0.21–0.75)
ICH volume > 100 ml	127	30.3		0.75 (− 0.15–0.91)
Homogenous ICH	1198	5.5		0.97 (0.92–0.99)
Heterogenous ICH	382	17.2		0.92 (0.53–0.97)

*ICH* Intracerebral hemorrhage, *IVH* Intraventricular hemorrhage, *IQR* Interquartile range, *LoA* Limits of agreement, *ICC* Interclass correlation, *CI* Confidence interval

### Agreement analysis between manual segmentation and the qER–NCCT volume for patients with IVH only

For the 44 patients with only IVH, there was excellent agreement between the two methods with an *ICC* of 0.95 (95% *CI*: 0.77–0.98). The Bland–Altman plot showed a significant difference in volume estimation between the methods (*MD* = 9 ml, *p* < 0.01), as seen in Table 2 and in the Bland–Altman plot (Fig. 4). The disagreement consisted mainly of underestimation by the qER–NCCT, compared to manual segmentation.

### Agreement analysis between manual segmentation and ABC/2 for isolated parenchymal ICH without IVH extension

In total, 891 patients with ICH without IVH extension were included in this analysis. The *ICC* was 0.97 (95% *CI*: 0.97–0.98), showing excellent agreement between the two methods. The Bland–Altman showed a significant difference (*p* < 0.01) in volumes between the methods (Fig. 4) that consisted mainly of overestimation by the ABC/2 method, compared to manual segmentation. The mean difference (*MD*) was 1.3 ml, and the 95% limits of agreement were (− 2–15), as shown in Table 2.

### Agreement between ABC/2 and qER–NCCT for ICH without IVH extension

In total, 836 patients with ICH without IVH extension were included in this analysis. There was good agreement between the two methods with an *ICC* of 0.86 (95% *CI*: 0.78–0.9). The Bland–Altman plot showed a significant difference in volumes (*p* < 0.05) that consisted mainly of volume underestimation by qER–NCCT, compared to the ABC/2 method (Fig. 4). The mean difference (*MD*) was 5.9 ml, and the 95% limits of agreement were (− 19 to 30). Excellent agreement with a volume difference ≤ 2 ml between qER–NCCT and ABC/2 was seen in 53% of the cases, of which 96% had a regular shape, and 85% were small ICH (≤ 10 ml), whereas a volume measurement difference of ≤ 2 ml was only seen in 1.5% of large (> 30 ml) heterogenous ICH. The best agreement between qER–NCCT and ABC/2 was seen in small (≤ 10 ml) hemorrhages (Fig. 4).

All included ICHs were subgrouped as supra- or infratentorial, and the supratentorial ICHs were further subdivided into lobar or deep. Bland–Altman plots were generated for each subgroup (Supplemental Figs. 2 and 3). In general, the agreement between the volumetric measurements was excellent or good in all subgroups, except for hematomas larger than 30 ml (Fig. 3). In lobar ICH, the qER–NCCT underestimated a portion of hematomas that typically were large and heterogenous in attenuation. A couple of typical examples are shown in Fig. 2, where it is evident that the software has delineated only the most hyperintense parts of the hematomas, thereby underestimating the total volume.

## Discussion

This study aimed to validate novel fully automated software developed by deep learning methodology (qER–NCCT), in measuring the volume of intracerebral hemorrhage on NCCT with manual segmentation as gold standard. The automated analysis was technically successful in returning a result to the PACS in 99.3% of all cases. The sensitivity for detecting ICH was 97%, and 54 of the 57 false negatives were 1 mm or smaller.

Our study showed excellent agreement (*ICC* = 0.96) between the ICH volumes obtained by qER–NCCT and manual segmentation in all patients, and good agreement (*ICC* = 0.86) between qER–NCCT and the ABC/2 method in patients with parenchymal ICH without IVH extension. For small (≤ 10 ml), homogenous and regular-shaped ICHs without intraventricular extension, the agreement was excellent between the two methods, and for ICHs between 10 and 30 ml, the agreement was good.

The significant volume difference between qER–NCCT and manual segmentation was mainly driven by underestimation in large (> 30 ml) heterogenous, irregular, or multilobulated supratentorial/lobar ICH and ICH with subarachnoid or intraventricular extension. The largest differences between qER–NCCT and manual segmentation were seen in cases such as the illustrative example in Fig. 2, where the software clearly is delineating the most hyper-intensive portions within the hematoma along the marked border to the portions of lower intensity. A likely explanation is that the deep learning algorithm used in this study is trained for segmenting homogenous hyperdense ICH. Underestimation by qER–NCCT was also seen in lobar ICH with multifocal ICH, fingerlike projections, and ICH with subarachnoid hemorrhage extension. Further development of the qER–NCCT tool should address this limitation; however, from a clinical perspective, the differences may have a limited impact in the group of ICH larger than 30 ml [23–26]. Since the qER–NCCT analysis precedes the assessment by the radiologist in a clinical implementation, such cases could easily be picked up and corrected by the radiologists, thereby adding the human quality for the small number of cases where the software yet is not sufficient by itself.

Heit et al. compared the automated ICH volume segmentation by the Rapid ICH module to manual segmentation and found a good correlation with a correlation coefficient of 0.983 [14]. Heit et al. included 158 patients with ICH, in contrast to our study which included a total of 1649 patients with ICH of varying sizes, attenuations, and shapes. Another automated ICH detection and volume measurement from Brainomix was evaluated by Schmitt et al. based on 160 NCCT with 0.91 sensitivity and 0.89 specificity for ICH detection and strong agreement (*ICC*: 0.98), compared to manual volume quantification [16]. Ironside et al. developed and validated a fully automated segmentation algorithm for volume quantification in 300 patients with supratentorial ICH. The algorithm showed a similar accuracy and improved workload efficiency, compared with manual volume segmentation methods [27]. Compared to these previous studies, our study is based on a much larger population and equally important is the fact that the population is selected based on a nationwide quality registry to ensure a very high coverage of the entire ICH spectrum.

The agreement between manual segmentation and qER–NCCT as well as with ABC/2 was best for small and homogenous ICH. This result is in line with a recent study by Delcourt et al. comparing ABC/2 to the MIStar software that also observed the largest differences in large and irregularly shaped ICH and ICH with subarachnoid extension [7], most often by volume overestimation by the ABC/2 method. Wang et al. showed similar overestimation by the ABC/2 method compared to a computer-assisted volumetric segmentation for large and irregularly shaped hematomas [28]. Contrarily, Maeda et al. found the ABC/2 method to systematically underestimate ICH volumes by 14.9%, compared to planimetric methods [9].

Scherer et al. also compared an automatic volume segmentation tool for ICH and showed that ABC/2 significantly overestimated the volume in large ICH, whereas the agreement was better for hematomas up to 40 ml [15].

The strengths of our analysis include the large dataset collected from the Swedish Stroke Register Riksstroke with systematic and consecutive data collection and imaging analysis. There were no imaging-based exclusions other than severe imaging artifacts; therefore, the dataset includes ICHs of varying sizes, shapes, and attenuation patterns, in addition to the presence of subarachnoid, subdural, and intraventricular extension.

Limitations of this study include the fact that only automated ICH volumetric software was evaluated, and further studies should compare the performance of several software in the same study population. Another limitation is that the ABC/2 analysis only was done in the portion of ICH without intraventricular extension.

## Conclusion

Our study showed excellent agreement between the fully automated ICH segmentation software (qER–NCCT) and manual segmentation in volume quantification of ICH on NCCT. The qER–NCCT would be an important additive tool for radiologists and clinicians by aiding in early diagnostics and prognostication for patients with ICH. The algorithm showed underestimation of ICH volume, mainly in large, heterogenous and irregularly shaped ICHs. Further refinement of the software should address this group; less precise measures are may be of less importance in very large hemorrhages. Since the qER analysis in a clinical setting would precede the manual assessment, such cases could be alerted for need of further analysis by the radiologists, thereby adding the human quality to the small number of cases where the software yet is not sufficient by itself and provide validated volumetry on a population-wide level.

## Supplementary Information

Additional file 1:  Supplementary Table 1: Interrater agreement for ICH volumes for manual segmentation and the ABC/2 method.
Supplemental Figure 1: Bland-Altman plot for the interrater agreement for manual segmentation, considered gold standard (left panel) and the ABC/2 method (right panel). The mean differences, the standard deviation and 95% Limits of Agreement is shown in each plot.
Supplemental Figure 2: Bland-Altman plots for the agreement between two methods for supratentorial ICH. Panel A shows the agreement between manual segmentation (gold standard) and the qER–NCCT software for all ICH. Panel B shows the agreement between manual segmentation (gold standard) and the qER–NCCT software for all supratentorial ICH. Panel C shows the agreement between manual segmentation (gold standard) and the ABC/2 method for supratentorial ICH without IVH. Panel D shows the agreement between the qER–NCCT software and the ABC/2 method for supratentorial ICH without IVH.Panel E shows the agreement between manual segmentation (gold standard) and the qER–NCCT software for all lobar ICH. Panel F shows the agreement between manual segmentation (gold standard) and the qER–NCCT software for all lobar ICH without IVH. Panel G shows the agreement between manual segmentation (gold standard) and the ABC/2 method for lobar ICH without IVH. Panel H shows the agreement between the qER–NCCT software and the ABC/2 method for lobar ICH without IVH. Panel I shows the agreement between manual segmentation (gold standard) and the qER–NCCT software for all deep ICH. Panel J shows the agreement between manual segmentation (gold standard) and the qER–NCCT software for all deep ICH without IVH. Panel K shows the agreement between manual segmentation (gold standard) and the ABC/2 method for deep ICH without IVH. Panel L shows the agreement between the qER–NCCT software and the ABC/2 method for deep ICH without IVH. The mean differences, the standard deviation and 95% Limits of Agreement is shown in each plot. Supplemental Figure 3: Bland-Altman plots for the agreement between two methods for Infratentorial ICH. Panel A shows the agreement between manual segmentation (gold standard) and the qER–NCCT software for all infratentorial ICH. Panel B shows the agreement between manual segmentation (gold standard) and the ABC/2 method for infratentorial ICH without IVH. Panel C shows the agreement between the qER–NCCT software and the ABC/2 method for infratentorial ICH without IVH. The mean differences, the standard deviation and 95% Limits of Agreement is shown in each plot.

## Abbreviations

AI: Artificial intelligence
CT: Computed tomography
*CI*: Confidence interval
DICOM: Digital imaging and communications in medicine
HU: Hounsfield units
ICH: Intracerebral hemorrhage
*ICC*: Intraclass correlation coefficient
IVH: Intraventricular hemorrhage
*IQR*: Interquartile range
*LoA*: Limits of agreement
*MD*: Mean difference
NCCT: Non-contrast computed tomography
PACS: Picture archiving and communication system
