# Supplementary material for: Accuracy of automated intracerebral hemorrhage volume measurement on non-contrast computed tomography: a Swedish Stroke Register cohort study
Source: Neuroradiology. 2022 Nov 3;65(3):479–88. doi: 10.1007/s00234-022-03075-9 (PMC9905189; doi:10.1007/s00234-022-03075-9)
Supplement: Supplementary file 1 — Supplementary Table 1: Interrater agreement for ICH volumes for manual segmentation and the ABC/2 method. Supplemental Figure 1: Bland-Altman plot for the interrater agreement for manual segmentation, considered gold standard (left panel) and the ABC/2 method (right panel). The mean differences, the standard deviation and 95% Limits of Agreement is shown in each plot. Supplemental Figure 2: Bland-Altman plots for the agreement between two methods for supratentorial ICH. Panel A shows the agreement between manual segmentation (gold standard) and the qER–NCCT software for all ICH. Panel B shows the agreement between manual segmentation (gold standard) and the qER–NCCT software for all supratentorial ICH. Panel C shows the agreement between manual segmentation (gold standard) and the ABC/2 method for supratentorial ICH without IVH. Panel D shows the agreement between the qER–NCCT software and the ABC/2 method for supratentorial ICH without IVH.Panel E shows the agreement between manual segmentation (gold standard) and the qER–NCCT software for all lobar ICH. Panel F shows the agreement between manual segmentation (gold standard) and the qER–NCCT software for all lobar ICH without IVH. Panel G shows the agreement between manual segmentation (gold standard) and the ABC/2 method for lobar ICH without IVH. Panel H shows the agreement between the qER–NCCT software and the ABC/2 method for lobar ICH without IVH. Panel I shows the agreement between manual segmentation (gold standard) and the qER–NCCT software for all deep ICH. Panel J shows the agreement between manual segmentation (gold standard) and the qER–NCCT software for all deep ICH without IVH. Panel K shows the agreement between manual segmentation (gold standard) and the ABC/2 method for deep ICH without IVH. Panel L shows the agreement between the qER–NCCT software and the ABC/2 method for deep ICH without IVH. The mean differences, the standard deviation and 95% Limits of Agreement is shown in each plot. Supplemen [file 234_2022_3075_MOESM1_ESM.docx]

# Supplementary Material

## Supplementary Table 1.

| Interrater agreement | Manual segmentation  Observer1 vs. Observer 2  n = 422  (all ICH) | ABC/2 method  Observer 1 vs. Observer 2  n = 252  (ICH without IVH) |
| --- | --- | --- |
| Mean difference (ml) | -0.35 | 0.31 |
| Standard deviation (ml) | 5.5 | 5.76 |
| Median difference (ml) | 0 | 2 |
| IQR (ml) | 3 | 2.5 |
| 95% LoA (ml, low – high) | -11 – 10.4 | -11 ^__^ 11.6 |
| ICC | 0.996 | 0.98 |
| 95% CI | (0.995- 0.996) | (0.96-0.99) |
| ICH= Intra Cerebral Hemorrhage, IVH=Intra Ventricular Hemorrhage, IQR=interquartile range, LoA=Limits of Agreement, ICC=Inter Class Correlation | | |

Supplementary Table 1. Interrater agreement for ICH volumes for manual segmentation and the ABC/2 method.

Supplemental Figure 1.


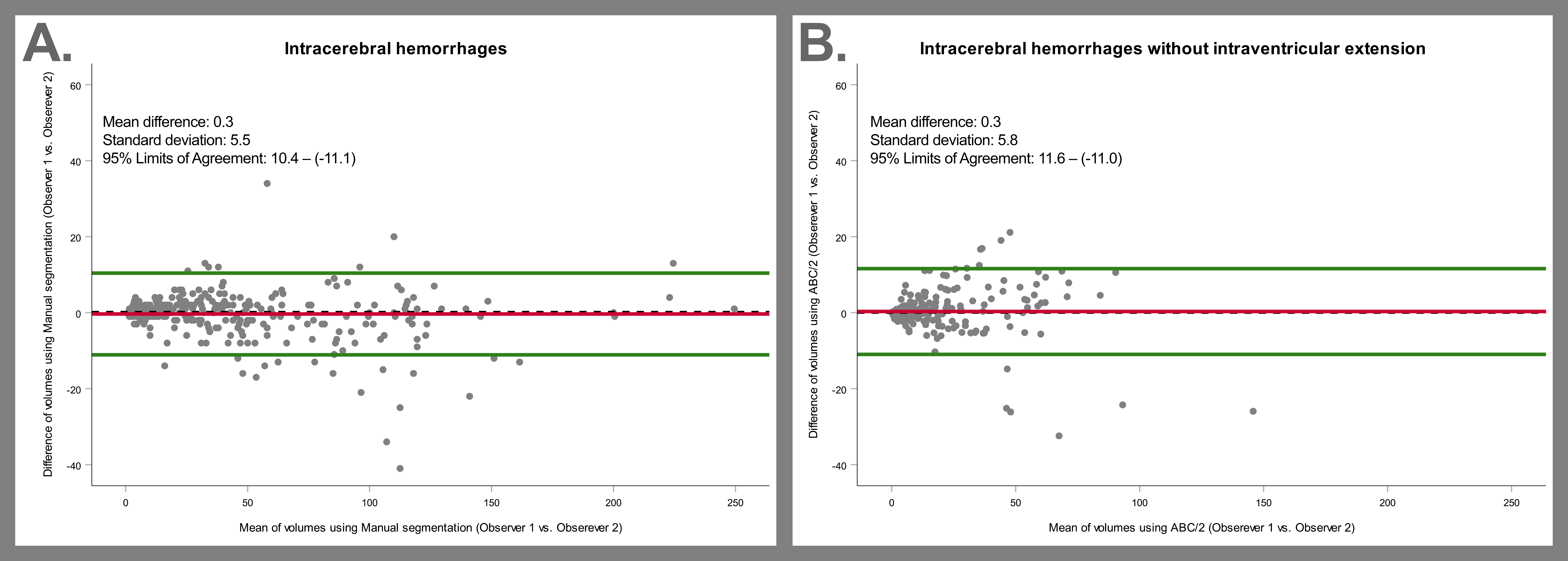


Supplemental Figure 1. Bland-Altman plot for the interrater agreement for manual segmentation, considered gold standard (left panel) and the ABC/2 method (right panel).

The mean differences, the standard deviation and 95% Limits of Agreement is shown in each plot.

Supplemental Figure 2.


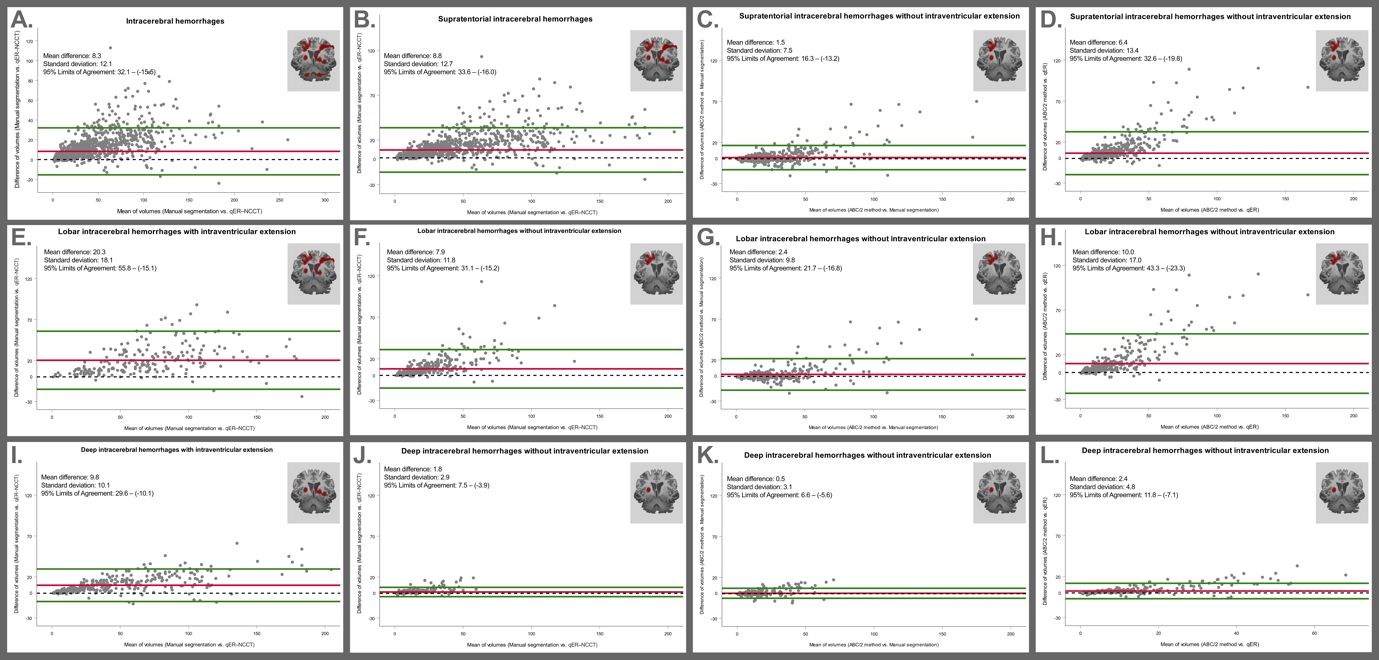


Supplemental Figure 2. Bland-Altman plots for the agreement between two methods for supratentorial ICH.

Panel A shows the agreement between manual segmentation (gold standard) and the qER–NCCT software for all ICH.

Panel B shows the agreement between manual segmentation (gold standard) and the qER–NCCT software for all supratentorial ICH.

Panel C shows the agreement between manual segmentation (gold standard) and the ABC/2 method for supratentorial ICH without IVH.

Panel D shows the agreement between the qER–NCCT software and the ABC/2 method for supratentorial ICH without IVH.

Panel E shows the agreement between manual segmentation (gold standard) and the qER–NCCT software for all lobar ICH.

Panel F shows the agreement between manual segmentation (gold standard) and the qER–NCCT software for all lobar ICH without IVH.

Panel G shows the agreement between manual segmentation (gold standard) and the ABC/2 method for lobar ICH without IVH.

Panel H shows the agreement between the qER–NCCT software and the ABC/2 method for lobar ICH without IVH.

Panel I shows the agreement between manual segmentation (gold standard) and the qER–NCCT software for all deep ICH.

Panel J shows the agreement between manual segmentation (gold standard) and the qER–NCCT software for all deep ICH without IVH.

Panel K shows the agreement between manual segmentation (gold standard) and the ABC/2 method for deep ICH without IVH.

Panel L shows the agreement between the qER–NCCT software and the ABC/2 method for deep ICH without IVH.

The mean differences, the standard deviation and 95% Limits of Agreement is shown in each plot.

Supplemental Figure 3.


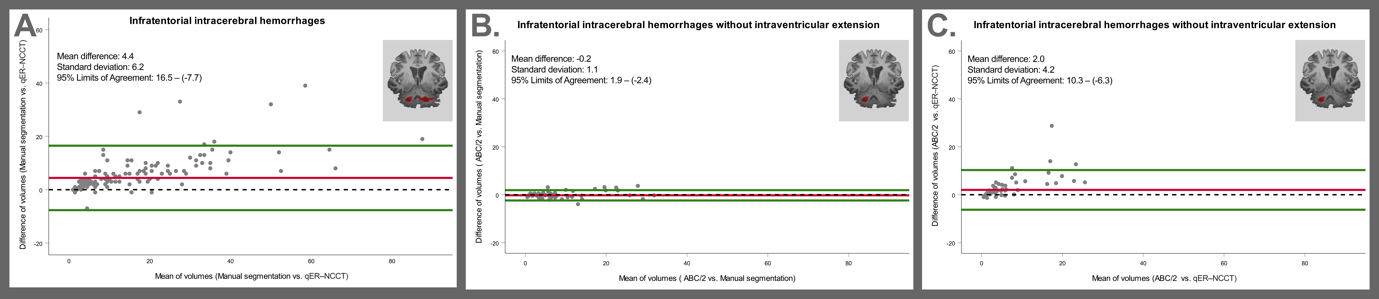


Supplemental Figure 3. Bland-Altman plots for the agreement between two methods for Infratentorial ICH.

Panel A shows the agreement between manual segmentation (gold standard) and the qER–NCCT software for all infratentorial ICH.

Panel B shows the agreement between manual segmentation (gold standard) and the ABC/2 method for infratentorial ICH without IVH.

Panel C shows the agreement between the qER–NCCT software and the ABC/2 method for infratentorial ICH without IVH.

The mean differences, the standard deviation and 95% Limits of Agreement is shown in each plot.
